# An Adversarial-Risk-Analysis Approach to Counterterrorist Online Surveillance

**DOI:** 10.3390/s19030480

**Published:** 2019-01-24

**Authors:** César Gil, Javier Parra-Arnau

**Affiliations:** 1 Mossos d’Esquadra, Catalan Police, 08080 Barcelona, Spain; cgile@uoc.edu; 2 Department of Computer Science and Mathematics, Universitat Rovira i Virgili, CYBERCAT-Center for Cybersecurity Research of Catalonia, 43007 Tarragona, Spain

**Keywords:** adversarial risk analysis, online surveillance, counterterrorism, threat identification, Internet of things

## Abstract

The Internet, with the rise of the IoT, is one of the most powerful means of propagating a terrorist threat, and at the same time the perfect environment for deploying ubiquitous online surveillance systems. This paper tackles the problem of online surveillance, which we define as the monitoring by a security agency of a set of websites through tracking and classification of profiles that are potentially suspected of carrying out terrorist attacks. We conduct a theoretical analysis in this scenario that investigates the introduction of automatic classification technology compared to the status quo involving manual investigation of the collected profiles. Our analysis starts examining the suitability of game-theoretic-based models for decision-making in the introduction of this technology. We propose an adversarial-risk-analysis (ARA) model as a novel way of approaching the online surveillance problem that has the advantage of discarding the hypothesis of common knowledge. The proposed model allows us to study the rationality conditions of the automatic suspect detection technology, determining under which circumstances it is better than the traditional human-based approach. Our experimental results show the benefits of the proposed model. Compared to standard game theory, our ARA-based model indicates in general greater prudence in the deployment of the automatic technology and exhibits satisfactory performance without having to relax crucial hypotheses such as common knowledge and therefore subtracting realism from the problem, although at the expense of higher computational complexity.

## 1. Introduction

The global threat of terrorism is currently one of the greatest challenges facing our society. Since 11 September, Western countries have been allocating more effort and resources to fight terrorism on the national and international scales. However, the resources for the increased security to counter potential large-scale attacks are limited.

In this context, the Internet is one of the most powerful means of propagating a threat with lethal effects, especially in the case of jihadist terrorism. In fact, a quantitative study [1] of 178 individuals detained in Spain between 2013 and 2016 for activities related to jihadist terrorism shows that there are two crucial factors for understanding their radicalization: (1) face-to-face or online contact with a radicalization agent; and (2) the existence of previous social links with other radicalized individuals. With the rise of the Internet of things (IoT), where billions of online objects embedded in our homes (e.g., smart grid technologies [2]), workplaces and cities will collect and analyze our data, the risk to national security is exacerbated while it opens up a new horizon for more invasive online surveillance technologies. We highlight, on the one hand, the emerging and recent deployment of vehicular ad hoc networks (VANETs) and specifically the vehicle cloud computing, a paradigm of cooperative mobile communications [3,4]. This type of networks represent a challenge in security and privacy since they require sophisticated mechanisms to protect against attacks coming from users connected to the network with identical privileges. On the other hand, wireless sensor networks are of crucial importance in the protection of critical infrastructures. The continuous monitoring of these infrastructures and the detection of malicious activity in the traffic of sensor networks requires advances that adapt to unknown attacks [5].

The revelations by NSA whistleblower Edward Snowden revealed the scale and extent of digital surveillance, particularly on the Internet, by different security and intelligence agencies [6]. In this work, we focus on the problem of *online surveillance* faced by a security agency that monitors a set of specific websites by tracking and classifying profiles that are potentially suspected of carrying out terrorist attacks. While there is an extensive body of research in decision-making models and risk analysis for fighting terrorism (We refer the reader to [7] for a complete review of the field.), to the best of our knowledge the problem above of online surveillance with counterterrorist purposes, understood as a game between opponents who want to maximize their benefits, has not been tackled yet. Although it is a controversial issue, our interest is to rationalize the matter from a strictly scientific point of view and, in any case, raise new questions and challenges.

The aim of this work is to conduct a theoretical analysis of the rationality conditions implied in the deployment of an online surveillance system for detecting and neutralizing potential terrorist threats on the Internet. We consider an approach for evaluating the problem based on adversarial risk analysis (ARA), whose bases are found in [8]. This approach supposes a new perspective of decision analysis, providing a robust analytical framework that is a hybrid between game theory and risk analysis. Its objective is to face precisely the risks derived from the intentional actions of intelligent adversaries, which increase security risks, and uncertain results.

We analyze the feasibility of using a technology based on an automatic suspect detection system that covers the functions of investigators who inspect certain websites. That is to say, we aim to determine under which circumstances the tracking and automatic detection model is better than the traditional model (“status quo”) in which the collected user profiles are inspected manually. Our work also allows us to limit the paradox of the false positive [9], which is a controversial problem of mass surveillance systems, since our approach is selective and does not infer errors from a broad reference population. Our objective is to carry out a rigorous analysis of the problem.

Next, we summarize the major contributions of this work:

- We analyze the suitability of decision-making models based on standard game theory and ARA, to tackle the problem of online surveillance. Our analysis contemplates the case of sequential defense-attack models, and examines the fulfillment of certain requirements on the defender and attacker’s side.
- We propose an ARA-based model to investigate the problem of online surveillance and analyze the rationality conditions of an automatic threat detection system. Our analysis constitutes a preliminary step towards the systematic application of ARA, in that it aims to establish a point of departure and connection between the analytical framework provided by ARA, a young field within risk analysis, and the problem of online surveillance.
- We conduct an experimental evaluation of the proposed decision-making model and illustrate the typical problem solving approach used in a real case. Our evaluation methodology, in fact, may serve as a template for real problems, which would basically add modeling and computational complexities. Furthermore, we carried out a sensitivity analysis and provide a thorough comparison with a standard game-theoretic approach under assumptions of common knowledge. Our experiments showed that our ARA-based model outperforms the standard game-theoretic approach, although at the expense of more costly solutions, from a computational point of view.
- The connection between the ARA models and online counterterrorism sheds new light on the understanding of the suitability of such decision-making models when it comes to applying them to the online surveillance problem. We also hope to illustrate the riveting intersection between the fields of ARA and threat intelligence, in an attempt towards bridging the gap between the respective communities.

The remainder of this paper is organized as follows. Section 2 provides some background on online third-party tracking and establishes our assumptions about the surveillance system. Section 3 describes the online surveillance problem tackled in this work. Section 4 examines the appropriateness of decision-making models based on standard game theory and ARA, to address the problem of online surveillance. Section 5 proposes an ARA-based model for sequential decision-making in the context of online surveillance. Section 6 conducts an experimental evaluation of the proposed model. Section 7 discusses several aspects of our model in relation to the experimental results. Finally, conclusions are drawn in Section 8.

## 2. Background and Assumptions

The purpose of online third-party tracking is behavioral advertising [10,11], that is to say, showing ads based on the user’s past browsing activity. In this section, we first give a brief overview of the main actors of the advertising ecosystem. This will be necessary to understand our assumptions about the online surveillance system assumed in this work, described below in Section 2.2.

### 2.1. Background in Online Third-Party Tracking

The online advertising industry is composed by a considerable number of entities with very specific and complementary roles, whose ultimate aim is to display ads on websites. Publishers, advertisers, ad platforms, ad agencies, data brokers, aggregators and optimizers are some of the parties involved in those processes [12]. Despite the enormous complexity and constant evolution of the advertising ecosystem, it is usually characterized in terms of publishers, advertisers and advertising platforms [13,14,15,16,17].

In this simplified albeit comprehensive terminology, the third-party tracking and advertising is carried out as follows. As users navigate the Web and interacts with websites, they are observed by both “first parties”, which are the sites the user visits directly, and “third parties”, which are typically hidden trackers such as ad networks embedded on most web pages. The former parties are often known as *publishers* and the latter as *ad platforms*.

Tracking by third-parties begins with publishers embedding in their sites a link to the ad platform(s) they want to work with. The upshot is as follows: when a user retrieves one of those websites and loads it, their browser is immediately directed to all the embedded links. Then, through the use of third-party cookies, web fingerprinting or other tracking technologies, the ad platform is able to track the user’s visit to this and any other site partnering with it. Third parties can learn not only the webpages visited and hence its content, but also the user’s location through their IP address, and, more importantly, their web-browsing interests, also known as *navigation trace*.

### 2.2. Assumptions

In this section, we describe our assumptions about the surveillance system deployed by a security agency for detecting possible terrorist threats on websites of interest. We acknowledge that a number of security and privacy aspects would need to be considered if such an online surveillance technology were to be deployed in real practice; among those aspects, the exchange of information between the security agency and the tracking/advertising platform(s) would be critical. However, the practical details of this system and possible anti-tracking countermeasures are beyond the scope of this work. The purpose of our analysis is not to explore these details but rather to study the rationality conditions of deploying such an online surveillance technology.

First, we suppose that a security agency wants to develop a web infrastructure on which to apply an online automatic threat detection system. The websites or publishers targeted by the agency will be those that make it possible to detect threats. For example, certain web forums where ISIS recruiting messages appear with certain frequency are sites that are susceptible to being investigated by the security agency.

In addition, we suppose that it is possible to track users’ activity in the target sites, or in other words, there are advertising and tracking companies operating on these sites. We acknowledge, however, that there may be sites such as those hosted on the dark web or others that are on the Internet that cannot be subject to surveillance because there are no ad platforms and tracking companies.

We assume that the agency can contract the services of the trackers available at the target sites to capture the users’ visit data, which may include, among others, their activity within the site, location, IP address and web-browser fingerprints. Once properly treated, all such data may allow the agency to re-identify a given web user, possibly with the help of the Internet service provider in question.

**Figure 1:**
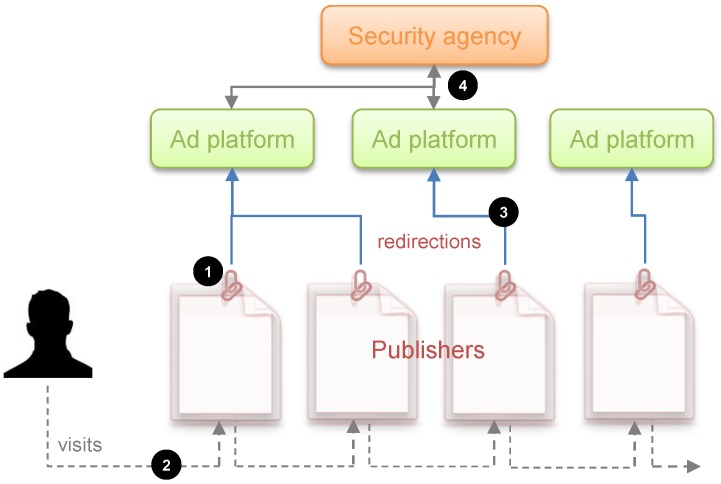
Third-party tracking requires that publishers include a link to the ad platform(s) they want to partner with (1). When a user visits pages partnering with this/these ad platform(s) (2), the browser is instructed to load the URLs provided by the ad platform(s). Through the use of third-party cookies and other tracking mechanisms, the ad platform(s) can track all these visits and build a browsing profile (3). Finally, the information collected by the ad platform(s) is shared with the security agency, provided that they have an agreement (4).

In essence, the infrastructure assumed is based on three well-differentiated activities. In a first stage, the agency selects its target publishers and hires the services of the companies that track them to obtain the users’ raw visit data. In a second optional stage, the agency exploits the data captured by the third-party trackers through an automatic system based on artificial intelligence methods (classifiers) so that, once the navigation trace of each user is extracted, it is possible to obtain a binary classification: suspicious or not suspicious. The threat detection algorithm that underlies this automatic system inevitably has certain sensitivity and specificity parameters (false positives). In a third and final stage, whether the automatic system has been deployed or not, there is an essential manual investigation of the flagged users by security experts. It should be noted that this type of architecture has two types of limited resources that are well differentiated: resources for hiring trackers and resources for the manual investigation of the collected profiles. In this work, we consider the cost of first type of resources is negligible compared to that of the latter. Figure 1 provides a conceptual depiction of the surveillance infrastructure assumed in this work.

## 3. The Problem of Online Surveillance

In this section, we describe the problem of online surveillance from the intrusion-detection problem posed and solved by [18] (According to [19]), intrusion detection systems (IDSs) are hardware or software systems that automate the process of monitoring events that have occurred in a computer system or network, analyzing them to detect security problems.. It is also appropriate to point out the work of Merrick and McLalay [20] on the use of scanners against smuggling of nuclear devices in cargo containers. Both works treat physical or logical security problems and assess their conditioning factors under uncertainty with the use of automatic threat detection systems. We rely on the cited works to define the problem at hand.

Suppose we are going to give support to the decision making of a security agency that has jurisdiction in a territory to prevent and neutralize attacks perpetrated by terrorists who use the Internet as a resource for carrying out their attacks. In general terms, we assume that the agency wishes to suffer the least possible harm, and, on the contrary, the terrorists want to cause the greatest possible damage. Faced with a normal Internet user, we define the suspect as a user whose digital activity can be considered a threat that must be investigated by the agency.

Suppose then that, in a certain period of time, the security agency will carry out online surveillance tasks on a series of websites that, based on expert knowledge, have been classified as susceptible to being used (propaganda, training, forums, etc.) by users who could potentially acquire the capabilities to prepare and/or carry out attacks.

To monitor these sites, the security agency uses a digital technology based on automatic detection of threats, described in Section 2, which consists of two well-defined complementary functions: automatic collection and classification of user profiles. Firstly, and based on tracking the digital activity of users who browse the target websites, the system collects the navigation traces, which result in unique user profiles. Secondly, of the profiles collected, the system is able to detect those that are potentially suspicious with certain sensitivity and specificity rates. More specifically, these classifiers are based on artificial intelligence methods. The use of the classifier is optional and in any case the system can always be supported by an “ad hoc” manual investigation by experts whose criteria we assume to be totally reliable. The classifier analyses each profile and if it considers it to be suspicious, it generates an alarm signal. Afterwards, the agency decides whether to investigate the profile based on available (limited) resources. Therefore, the agency makes decisions about whether to investigate according to the state (signal or lack of signal) of the system. However, when the system generates a signal, the agency does not know with certainty whether it is a real threat or the system has generated a false alarm. On the other hand, the suspect user’s main objective is not to be detected by the surveillance system, which would imply, immediately and to simplify, the success of their actions.

The aim of the agency is to configure the system by choosing a point in its effectiveness function that minimizes the total cost of surveillance (the cost is not necessarily a monetary value but we can treat values such as image, privacy, etc., or in any case monetize them). Thus, we initially define the probability of detection α as the probability of classifying a suspect conditioned on the user really being a suspect, and the probability of a false positive β as the probability of classifying a suspect conditioned on the user not being a suspect. In a perfect surveillance system, we would suppose α=1 and β=0. However, and in general, online surveillance technology is such that a high value of α also implies a high value of β, due to the variability of the data associated with the normal and abnormal traces and the imprecision of the algorithms used by these types of systems.

In general terms, the navigation trace of potential suspects will depend on factors such as the benefit derived from acquiring the capacities to carry out terrorist acts of different levels; the loss that they will receive if they are captured; and the probability that they will be detected. We assume that a potential terrorist obtains a benefit *b* if their navigation is not detected. If it is detected, the user incurs a loss *l* over a non-positive net benefit of (b−l)⩽0. Suppose that it is reasonable to think that l=(1+λ)b, with λ⩽0. The loss can take different forms depending on the nature of the terrorist potential (cost of legal persecution, reputation, intimidating effect, etc.). We denote by π the probability of the presence of a suspicious user in the set of monitored sites.

The agency complements the system with a manual investigation conducted by security experts. In general, it is expensive to always carry out manual investigations (it is obvious that it is a limited resource). When the agency does not deploy the automatic system, expert investigators must manually investigate a proportion ρ of the user profiles collected. When the system is deployed, experts can only investigate a proportion $\rho_{1}$ of the profiles that generated alarm signals and a proportion $\rho_{0}$ of the profiles that did not generate signals. The agency incurs a cost *c* every time the experts conduct a manual investigation. We assume that expert manual investigation always confirms or discards threats with certainty (it is 100% effective). If the agency detects a threat it will not incur any loss other than the cost *c* of the manual investigation. When a suspicious profile is not detected, the agency incurs a damage *d*. Suppose again that it is reasonable to think that c⩽ϕd, with ϕ⩽1. It is usual to estimate these possible damages in the risk assessment phase before implementing and configuring the detection system. Traditionally, the quality function of a detection system is modeled through its relative operating characteristic (ROC) curve, although other evaluation functions can be appropriate, as shown in the next section. Table 1 summarizes the parameters of the problem of online surveillance defined in this section.

## 4. Analysis of Decision-Making Models

The terrorist attacks that occurred in Western countries in the last decades have sparked a growing interest in decision-making models and risk analysis for fighting terrorism. We refer the reader to the work in [7] for a complete review of the field.

The vast majority of this literature adopts a game-theoretic approach [21]. For example, [22], studied multi-attribute utility functions for the defender and attacker, and for simultaneous and sequential actions, to compute Nash equilibria; and [23] proposed several max-min optimization models to tackle defender-attacker, attacker-defender and defender–attacker–defender–problems. A hybrid model between game theory and risk analysis is ARA [8], a new perspective of decision analysis that differs from standard game theory in that it makes no assumptions of common knowledge.

The other mainstream literature adopts a decision-analysis approach. Among such works, we highlight the work in [24], which uses decision trees to assess man-portable air defense systems countermeasures. The recurrent problem of decision analysis, however, is the need to evaluate the likelihood of the actions of the others, which is a central issue of the Bayesian approach to games (We would like to stress that the tension between game-theoretic and decision-analytic approaches to decision-making problems with adversaries is by no means exclusive of counterterrorism models [21].).

This section is organized as follows. We first specify our requirements for the desired decision-making model in Section 4.1. Then, Section 4.3 examines the classical game-theoretic approach, and Section 4.4 analyzes ARA and verifies whether the requirements are met by these two models.

### 4.1. Model Requirements and Notation

In this section, we focus on standard game theory and ARA, and analyze their suitability to tackle the online surveillance problem described in Section 3. Since the aim of our analysis is to gain insight into the rationality of online surveillance with the principle of being as close as possible to reality, we define the following requirements for such a model:

- Both opponents (intelligent, rational) want to maximize their utility.
- There is uncertainty about the attacker’s actions due to uncertainty about their utilities and probabilities.
- The information on the evaluation of the objectives between opponents is incomplete, with the possibility of obtaining it partially through different sources that we will call intelligence (experts, historical data and/or statistical distributions).
- And it is possible to model simultaneous and non-simultaneous (sequential) decisions.

Throughout this section, we follow the convention of using uppercase letters for random variables (r.v.s), and lowercase letters for the particular values they take on. Accordingly, $\hat{p}$ denotes approximation, estimation, as a result of Monte Carlo simulation; and $p^{k}∼P$ denotes the former is the *k*th iteration of the Monte Carlo simulation of the latter r.v. In the text, we drop the superindex *k* for notational simplicity.

### 4.2. Sequential Defense–Attack Model

To study the appropriateness of standard game theory and ARA, we develop first the sequential defense–attack model, which is one of the two standard counterterrorism model (Other standard models include the simultaneous defense-attack model, the sequential defense–attack–defense [25] and the sequential defense-attack with private information.). We use this model to analyze the problem that is the objective of this work. For the sake of comparison, we consider the following example of counterterrorism scenario.

**Example 1** (Counterterrorism scenario)**.** *The authority of an airport (D, the defender) must decide whether to install body scanners at the security checkpoints of an airport, replacing the X-ray scanners. On the other hand, a terrorist group (A, the attacker) decides whether to try to smuggle a bomb onto an airplane. D makes the first move, so A can see if the new body scanners are in use when they arrive at the airport. Because A can observe the actions of D before deciding their move, they do not need to know their probabilities or utilities. However, D must have a distribution for A, which specifies its utilities and probabilities.*

In this model, the defender makes the first move, deploys their defensive resources and makes a certain choice to position themselves against the possible terrorist threat. The attacker, after having observed this decision, evaluates their options and carries out an attack.

We assume that the defender initially has a discrete set of possible decisions $D=\{d_{1},d_{2},\ldots ,d_{m}\}$ and that the attacker can respond with one of their possible attacks $A=\{a_{1},a_{2},\ldots ,a_{p}\}$. As a consequence of these actions, a result is produced. This result is the only uncertainty of the problem and depends probabilistically on $\left(d,a\right)\in D\times A$. The decision sets can include the option to do nothing or combine several defenses or several attacks. To simplify the discussion, we consider only two possible values for the result, S={0,1}, which represents the failure or success of the attack. Thus, the defender and the attacker can have different probability distributions for the possibility of success, given a pair $\left(d_{i},a_{j}\right)$. They can also have different utility functions.

To visualize the situation, we have built the influence diagram and the decision tree corresponding to the problem at hand. These are two decision analysis tools that help us to gain a clearer view of the sequential decisions that have to be made.

An influence diagram is a directed acyclic graph with three kinds of nodes: decision nodes, which are shown as squares; chance or uncertainty nodes, shown as circles; and value nodes, shown as hexagons. In addition, an influence diagram can have three types of arcs depending on their destination: if the arc arrives at a decision node, this indicates that the decision is made knowing the value of the predecessor; if it arrives at a random node, then the uncertainty depends on the predecessor node conditioned probability; and if it arrives at a value node, then the utility reflected in that value node depends on the values of the predecessors.

**Figure 2:**
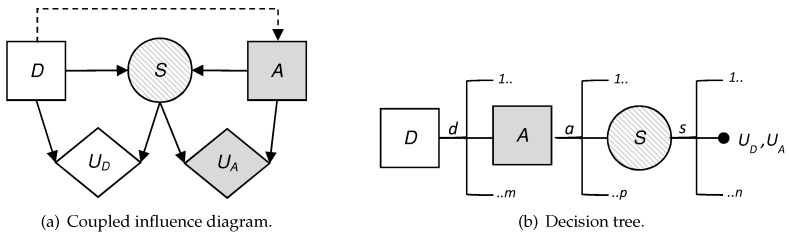
Sequential defense–attack model.

Figure 2 shows the coupled influence diagram of the model. In view of this, we assume that the consequences for the defender and the attacker depend, respectively, on (d,s) and (a,s). This is then inferred to the defender’s utility node by two arcs coming from the decision node, *D*, and the uncertainty node, *S*, which represents the result. The decision node representing the attacker’s utility also has two arcs, which in this case come from the decision node *A* and the uncertainty node *S*. It is also reflected that the result node, *S*, depends, in this case probabilistically, on the defender’s initial action and the attacker’s response. The influence diagram arc from the node of the defender’s first decision to the attacker’s node reflects that the defender’s choice is observed by the attacker before they decide on their attack.

We also show in Figure 2b the decision tree for this problem, clearly reflecting its sequential nature. First, a decision is made corresponding to the set *D*; once the attacker observes this descision, they decide whether to attack; the final result is produced as a consequence of these two actions. Note that there are two utility values in the terminal node of the tree. Each of these represents the utility that corresponds to each of the actors: one value refers to the defender’s utility and the other value refers to the attacker’s utility. The fact that there are several branches of each of the nodes refers to the possible decisions or results, which is the case of the chance node, which can be taken in each of them. The number of possible decisions in each decision node is not always the same and that is reflected in the decision tree.

### 4.3. Analysis Based on Standard Game Theory

The focus of game theory to solve the posed problem requires obtaining the utility functions of the defender $u_{D}\left(d,s\right)$ and attacker $u_{A}\left(a,s\right)$, as well as evaluating the probability of the event S|d,a for each of the participants, which we designate as $p_{D}\left(S|d,a\right)$ and $p_{A}\left(S|d,a\right)$ for the defender and the attacker, respectively. Standard game theory requires as initial assumption that the defender knows the attacker’s utilities and probabilities and the attacker knows the defender’s utilities and probabilities, this being *common knowledge*. If this happens, a solution to the problem can be obtained from the decision tree (Figure 2b) by backward induction as follows.

In node *S*, it is common knowledge for the two participants that the defender’s expected utility associated with each pair $\left(d,a\right)\in D\times A$,

(1) $$\psi_{D}\left(d,a\right)=p_{D}\left(S=0|d,a\right)u_{D}\left(d,S=0\right)+p_{D}\left(S=1|d,a\right)u_{D}\left(d,S=1\right),$$

and the attacker’s expected utility associated with $\left(d,a\right)\in D\times A$,

(2) $$\psi_{A}\left(d,a\right)=p_{A}\left(S=0|d,a\right)u_{A}\left(d,S=0\right)+p_{A}\left(S=1|d,a\right)u_{A}\left(d,S=1\right).$$

Knowing what the defender will do in decision node *D*, the attacker can determine what is their best attack in node *A*, after observing the defensive action of the defender, solving the problem

(3) $$a^{*}\left(d\right)={arg max}_{a\in A}\;\psi_{A}\left(d,a\right),\forall \;d\in D.$$

This is also known by the defender due to the hypothesis of common knowledge. The defender can determine their best decision in the decision node *D*, solving the problem

(4) $$d^{*}={arg max}_{d\in D}\psi_{D}\left(d,a^{*}\left(d\right)\right).$$

Thus, under the assumption of common knowledge, standard game theory predicts that the defender will choose $d^{*}\in D$ in node *D*. Then, the attacker will respond by choosing the attack $a^{*}\left(d^{*}\right)$. The pair $\left(d^{*},a^{*}\left(d^{*}\right)\right)$ determines a solution of the game and is a Nash equilibrium.

### 4.4. Analysis Based on ARA

Now we abandon the assumption of common knowledge. It should be taken into account that ARA serves here as support to the defender.

**Figure 3:**
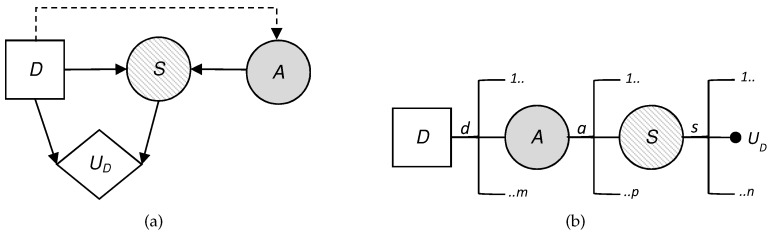
(**a**) Influence diagram of the defender and (**b**) decision tree of the defender.

To do this, we treat the attacker’s behavior in node *A* as uncertainty from the defender’s point of view and we model this added uncertainty. This is reflected in the influence diagram and the decision tree, as the attacker’s decision node has become a chance node, replacing the square by a circle. Looking at the influence diagram (Figure 3a) we now need to obtain $p_{D}\left(A|d\right)$, the probability that the defender will assign to the attack what the attacker will choose once they have observed every defensive move d∈D of the defender. The defender also needs to evaluate $u_{D}\left(d,s\right)$ and $p_{D}\left(S|d,a\right)$, already described above.

Once these data have been evaluated, the defender can solve their decision problem with backward induction considering the decision tree (Figure 3b). Then, the defender will obtain their expected utility in the node *S*, $\psi_{D}\left(d,a\right)$, for each pair $\left(d,a\right)\in D\times A$ in the same way as in the previous approach. It is at this time when the defender can use the evaluation of the probability of what the attacker will do faced with each of the defender’s decisions, $p_{D}\left(A|d\right)$, to determine their expected utility in the node *A* for each d∈D, with the expression

(5) ψDd=∑i=1pψDd,aip^DA=aiA=aidd.

Finally, the defender can find the decision that maximizes their expected utility in node *D*, solving the problem

(6) $$d^{*}={arg max}_{d\in D}\psi_{D}\left(d,a^{*}\left(d\right)\right).$$

Therefore, the best strategy for the defender for the defense–attack model is to choose first $d^{*}$ in node *D* after having observed s∈S.

**Figure 4:**
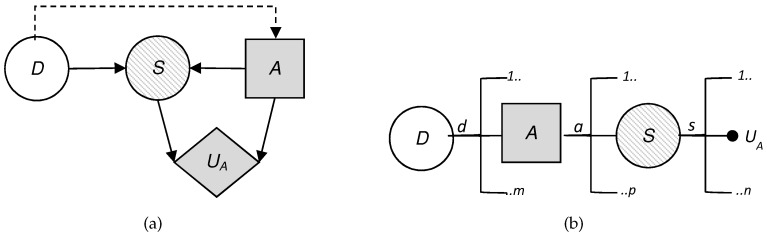
(**a**) Influence diagram of the attacker and (**b**) decision tree of the attacker.

The key now is how to evaluate $p_{D}\left(A=a|d\right)$. To do this, ARA assumes the defender can use a statistical method if they have historical data on the attacker’s behavior in similar situations. To complement this evaluation, the defender could also use expert opinions. However, as we describe in Section 5, an approach could be modeling the uncertainty that the defender has about the attacker’s decision. This can be done assuming: (i) the attacker wants to maximize their expected utility; and (ii) the defender’s uncertainty in evaluating this probability stems from the uncertainty that the defender has about the attacker’s probabilities and utilities associated with the attacker’s decision problem. In short, the evaluation is limited to analyzing the attacker’s decision problem from the defender’s point of view (Figure 4). The evaluation of the attacker’s probabilities and utilities from the defender’s perspective will be based on all the information that the defender has available, which can include previous data from similar situations and expert opinions. If the defender does not have this kind of information, they can use an uninformative or reference distribution to describe pDA=aA=add. Therefore, to obtain pDA=aA=add, the defender needs to evaluate $u_{A}\left(a,s\right)$ and $p_{A}\left(S|d,a\right)$, the attacker’s utilities and probabilities, which are unknown to the defender.

If the defender can access the attacker’s probabilities and utilities they will learn, by the same procedure as in the game theory approach, the action that the attacker would give most expected utility, $a^{*}\left(d\right)$, for each d∈D, and therefore, pDA=a∗(d)A=a∗(d)dd=1. This would imply that the attacker’s decision would be anticipated by the defender, and therefore they would not need to evaluate pDA=aA=add.

We start, therefore, from the assumption that the defender does not know these two quantities, but can recognize their uncertainty about them by means of a probability distribution $F=(U_{A}\left(a,s\right),P_{A}\left(S|d,a\right))$ and solve the attacker’s decision problem using backward induction on the decision tree of Figure 4b with the expression

(7) ΨAd,a=PAS=0S=0d,ad,aUAa,S=0+PAS=1S=1d,ad,aUAa,S=1.

In node A, assuming that the attacker wants to maximize their expected utility, the defender’s distribution on the attacker’s choice when the defender has considered their defense *d* is

(8) $$p_{D}\left(A=a^{*}|d\right)=P_{F}\left[a^{*}={arg max}_{a\in A}\Psi_{A}\left(d,a\right)\right].$$

This distribution can be approximated using Monte Carlo simulation methods, generating *n* values, such that

(9) p^DA=aA=add=|{a=arg maxx∈AψAi(d,x)}|/n,∀a∈A.

Once the defender has completed their evaluations, from these data they can solve their problem in the S node for each $\left(d,a\right)\in D\times A$ with the expression

(10) ΨDd,a=pDS=0S=0d,ad,auDd,S=0+pDS=1S=1d,ad,auDd,S=1.

Then, their estimated expected utility is

(11) ψ^Dd=∑i=1kψDd,aip^DA=aiA=aidd

and finally their optimal decision is

(12) $$d^{*}={arg max}_{d\in D}{\hat{\psi }}_{D}\left(d\right).$$

In view of our analysis of standard game theory and ARA, we regard the latter as the most appropriate model for evaluating the online surveillance problem defined in Section 3. The counterterrorism modeling based on common-knowledge assumptions entails that parts have too much information about their counterparts, which does not seem to make sense in a field in which secrecy tends to be an advantage. In a scenario where the adversary wishes to increase the risks of the defender, it seems unusual that the defender will have a full knowledge of their objectives, intentions or possible movements. Similarly, it seems unrealistic that the adversary fully knows the objectives, intentions or possible movements of the defender. Table 2 summarizes the analysis of the two models by matching the initial requirements defined in Section 4.1. As we show in next section, we rely on the analyzed ARA model to tackle the problem at hand.

## 5. An ARA Model for the Online Surveillance Problem

We present an ARA model to evaluate the problem of online surveillance described in Section 3. This model allows us to analyse the rationality conditions of the automatic threat detection system.

We assume that we support an agent (the agency, the defender, *D*) in their decision-making in relation to deploying an online surveillance system to monitor a set of selected websites, faced with the threat posed by the presence of the other agent (the suspect, the adversary, *A*) in the target sites. We assume that both agents operate monolithically.

**Figure 5:**
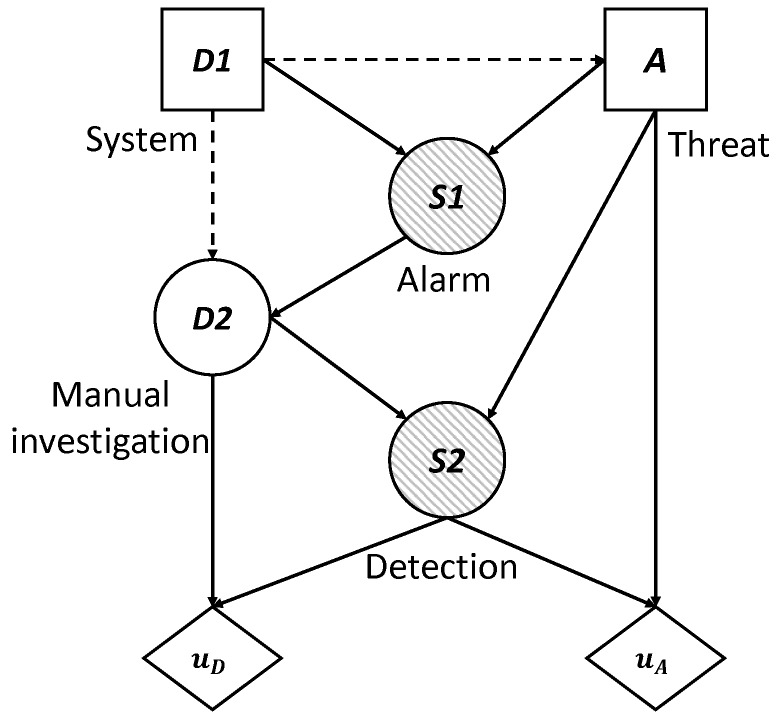
Influence diagram for the online surveillance problem.

According to the premises described in Section 2, we assume that the dynamics of the defender and the adversary can be described by means of a sequential defense–attack decision model represented in Figure 5 as an influence diagram coupled for the two agents.

To begin and given a set of target websites, the defender makes his initial decision $d_{1}=\left\{0,1\right\}$ (0 is No, 1 is Yes) about using the technology, represented by the decision node $D_{1}$. The adversary knows about these tracking measures and even so decides to be present in the set of sites, a={0,1}, represented by the decision node *A*. The automatic system, in the case that it is deployed ($d_{1}=1$), can lead to a system alarm signal, $s_{1}=\left\{0,1\right\},$ represented by the node of uncertainty $S_{1}$ shared by the defender and the adversary (if the system is not deployed, $s_{1}=0$ unfailingly). Depending on the previous result, the defender manually investigates the alarm, $d_{2}=\left\{0,1\right\}$, represented by the node of uncertainty $D_{2}$, to the degree that their (limited) resources allow. All this leads to the final result of the success/failure of the two agents, $s_{2}=\left\{0,1\right\}$, represented by the node of uncertainty $S_{2}$. We understand as success for the security agency the fact of detecting the threat and avoiding its potential actions, and failure is understood as the opposite. For the adversary, success and failure are the reverse events of the defender.

The utility $u_{D}$ obtained by the defender depends on the added cost of the manual investigation and the final success of the surveillance (nodes $D_{2}$ and $S_{2}$), on which their utility function is applied. Similarly, the utility $u_{A}$ obtained by the attacker depends on the added cost of access to the set of sites and the final success of the surveillance (nodes *A* y $S_{2}$), on which their utility function is applied.

### 5.1. The Defender’s Decision Problem

**Figure 6:**
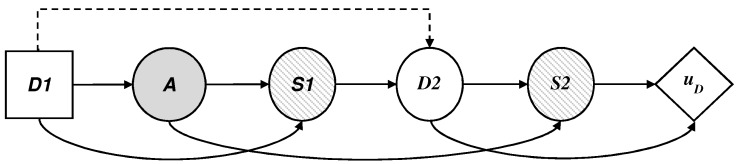
Influence diagram for the defender’s decision problem.

We describe in this section the defender’s decision problem, illustrated by an influence diagram in Figure 6, where the threat appears as a probability node A, from the point of view of *D*, which, given a collected profile should:

- decide if they use the technology, assigning values $d_{1}=\left\{0,1\right\}$ in node $D_{1}$.
- face the possible existence of a threat a={0,1} in node *A*.
- observe optionally, given the case, the result of the automatic detection system, $s_{1}=\left\{0,1\right\}$, in node $S_{1}$.
- establish proportions of profiles investigated manually based on the available resources, assigning values $d_{2}=\left\{0,1\right\}$ in node $D_{2}$.
- observe the final result of the surveillance,${\;s}_{2}=\left\{0,1\right\}$, in node $S_{2}$; and.
- add their costs and evaluate the results with their utility function $u_{D}$.

To solve the decision problem, *D*, it is necessary to evaluate the probability distributions, $p_{D}\left(A|d_{1}\right)$, $p_{D}\left(S_{1}|a,d_{1}\right)$, $p_{D}\left(D_{2}|d_{1},s_{1}\right)$ and $p_{D}\left(S_{2}|a,d_{2}\right)$ and the utility function $u_{D}\left(d_{2},s_{2}\right)$. Assuming that *D* is capable of providing such inputs, we would proceed by applying standard decision analysis calculations based on dynamic programming to obtain the optimal decision.

1. First, for each relevant scenario ($d_{2},s_{2})$, add the consequences and obtain the utility $u_{D}\left(d_{2},s_{2}\right)$.
2. In node $S_{2}$, calculate the expected utilities:
  (13) ψDd1,s1,d2=∑s2uDd2,s2pDS2S2d2,ad2,a.
3. In node $D_{2}$, calculate the expected utilities:
  (14) ψDd1,s1=∑d2ψDd1,s1,d2pDD2D2d1,s1d1,s1.
4. In node $S_{1}$, calculate the expected utilities:
  (15) $$\psi_{D}\left(d_{1},a\right)=\sum_{s_{1}}\psi_{D}\left(d_{1},s_{1}\right)\;p_{D}\left(S_{1}|d_{1},a\right).$$
5. In node *A*, calculate the expected utilities:
  (16) ψDd1=∑d2ψDd1,apDAAd1d1.
6. Finally, the decision node $D_{1}$ maximizes the expected utility and stores the corresponding optimal initial decision $d_{1}^{*}$.
  (17) $$\psi_{D}=\underset{d_{1}}{arg max}\psi_{D}\left(d_{1}\right).$$

Then, $d_{1}^{*}$ describes the optimal decision for the defender.

It should be kept in mind that we can describe the defender’s optimization problem with the expression

(18) $$d_{1}^{*}=\underset{d_{1}}{arg max}\sum_{a}\sum_{s_{1}}\sum_{d_{1}}\sum_{s_{2}}u_{D}\left(d_{2},s_{2}\right)p_{D}\left(S_{2}|d_{2},a\right)p_{D}\left(D_{2}|d_{1},s_{1}\right)p_{D}\left(S_{1}|d_{1},a\right)p_{D}\left(A|d_{1}\right).$$

Note that of the four values required by the agency, $p_{D}\left(A|d_{1}\right)$ is the most problematic, insofar as it involves the defender’s beliefs about the adversary’s decision once they have observed the defender’s initial decision $d_{1}$. This is an evaluation that requires strategic thinking for which we propose an approach based on ARA. For this, we need to solve the adversary’s decision problem, assuming uncertainty about their evaluations and propagating it to obtain its expected distribution based on the optimal presence of the adversary in the set of monitored sites. We discuss this in the following section.

### 5.2. The Adversary’s Decision Problem

**Figure 7:**
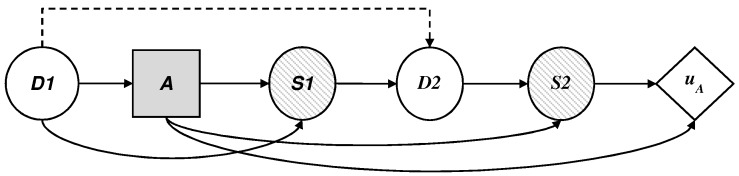
Influence diagram for the adversary’s decision problem.

We describe the dynamics of the threat, illustrated as an influence diagram in Figure 7, according to the defender’s point of view, where $D_{1}$ is now a probability node for the attacker, who must:

- observe the initial decision of *D*, $d_{1}=\left\{0,1\right\}$.
- decide on their presence in the set of monitored sites, a={0,1}, with impact over time if they are not detected.
- observe their success $s_{2}=\left\{0,1\right\}$ after the defender makes their allocations $d_{2}=\left\{0,1\right\}$ on the manual investigation of the profiles; and
- add their costs and obtain the corresponding utility $u_{A}$.

To solve the decision problem, we assume that the adversary wants to maximize their expected utility. They therefore need to evaluate $p_{A}\left(S_{1}|a,d_{1}\right)$, $p_{A}\left(D_{2}|s_{1},d_{1}\right)$, $p_{A}\left(S_{2}|d_{2},a\right)$, and $u_{A}\left(a,s_{2}\right)$. We cannot easily obtain these values so we model the defender’s uncertainty about them with random probability distributions. Then, we can propagate this uncertainty using the standard reduction algorithm of influence diagrams and obtain the optimal and random decision a={0,1} for each value of $d_{1}=\left\{0,1\right\}$. This provides us with the required distribution pDAAd1d1=P(A∗(d1)=a).

1. Add the consequences and obtain the random utility $U_{A}\left(a,s_{2}\right)$, for each (*a*, $s_{2}$).
2. In node $S_{2}$, calculate the expected random utilities:
  (19) ΨAa,s1,d2=∑s2UAa,s2PAS2S2d2,ad2,a.
3. In node $D_{2}$, calculate the expected random utilities:
  (20) ΨAd1,a,s1=∑d2ΨAa,s1,d2PAD2D2d1,s1d1,s1.
4. In node $S_{1}$, calculate the expected random utilities:
  (21) ΨAd1,a=∑s1ΨAd1,a,s1PAS1S1d1,ad1,a.
5. In node *A*, calculate the (random) optimal decision in response to each value of $d_{1}$:
  (22) $$A^{*}\left(d_{1}\right)={arg max}_{a}\Psi_{A}\left(d_{1},a\right).$$

This provides us with the required distribution $p_{D}\left(A=a^{*}|d_{1}\right)=P\left[A^{*}\left(d_{1}\right)=a\right]$, assuming that the space of *a* is discrete. It should be kept in mind again that the reduction of the previous influence diagram can be recast as

(23) $$A^{*}\left(d_{1}\right)=\underset{a}{arg max}\sum_{s_{1}}\sum_{d_{2}}\sum_{s_{2}}U_{A}\left(a,s_{2}\right)P_{A}\left(S_{2}|d_{2},a\right)P_{A}\left(D_{2}|d_{1},s_{1}\right)P_{A}\left(S_{1}|d_{1},a\right).$$

The distribution $p_{D}\left(A|d_{1}\right)$ can be estimated by Monte Carlo simulation. To do this, we sample *n* times the probabilities and utilities of the set

(24) $$F=\{P_{A}\;\left(S_{1}|a,d_{1}\right),P_{A}\;\left(D_{2}|s_{1},d_{1}\right),P\left(S_{2}|d_{2},a\right),U\left(a,s_{2}\right)\}$$

to obtain the optimal decision $a^{*}∼A^{*}\left(d_{1}\right)$ in the k-th iteration of the Monte Carlo simulation, k=1,…,n. Then, we can approximate $p_{D}\left(A|d_{1}\right)$ through $|1⩽k⩽n:\;a_{k}^{*}=a|/n$.

Note that, of the four components in *F*, the first three can be easily obtained. Normally, $P_{A}\left(S_{1}|a,d_{1}\right)$ would be related to $p_{D}\left(S_{1}|a,d_{1}\right)$ through some uncertainty, as we are referring to the results and the interaction between the attacker and defender, based on their decisions $d_{1}$ and *a*. This is also true for $P_{A}\left(S_{2}|d_{2},a\right)$ with respect to $p_{D}\left(S_{2}|d_{2},a\right)$. Regarding $U_{A}$, we generally have information about the multiple interests of the adversary, which we add. However, the fourth element, $P_{A}\left(D_{2}|s_{1},d_{1}\right)$, could require strategic thinking. In fact, the proposal presented here can be seen as a model of “level-2” thought, in which the defender optimizes their expected utility, with adverse probabilities derived from the optimization of the expected utilities (at random) of the adversary (Algorithm 1).

**Algorithm 1:** Overall attacker–defender approach.

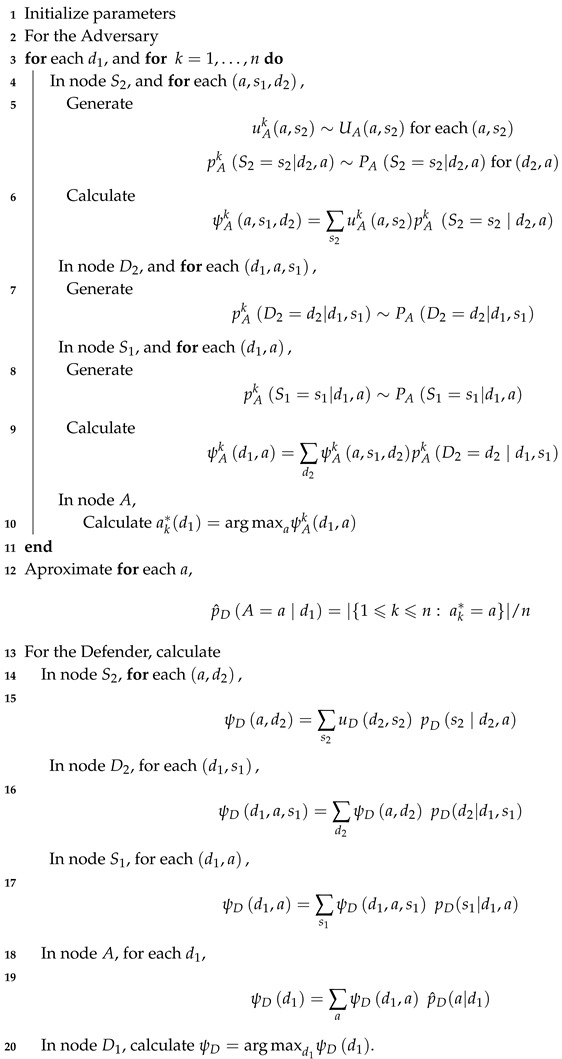



### 5.3. Overall Approach

The above ideas can be integrated into a step-by-step algorithm. First, we use simulation to estimate the distribution that predicts the options of the adversarial (suspicious) presence in the monitored sites. Second, we find the optimal initial allocation $d_{1}^{*}$ (in favor or against the deployment of technology), maximizing the defender’s expected utility with respect to the distribution of the derived prediction. We assume that the intervening r.v.’s are discrete, that is, that the impacts $S_{1}$ and $S_{2}$ are classified.

In the previous scheme, $d_{1}$ would be the optimal initial defense allocation. The corresponding probabilities of adversary presence, $p_{D}\left(A=a|d_{1}\right)$, represent the probability of each adversary scenario after deploying $d_{1}^{*}$, which would help raise awareness about the state of the security.

It is important to take into account that we make simulations for all possible initial allocations $d_{1}$. Our problem is a binary allocation on using a technology or continuing with the status quo; however, in problems where the number of these allocations/resources is too large, we could use a regression meta-model, as explained in [26], simulating some defenses, evaluating the corresponding attack probabilities and, consequently, approaching the attack probabilities in other defenses. Then, we would use that estimated attack prediction distribution to find the optimal resource allocation.

## 6. Experimental Evaluation

This section evaluates the decision-model proposed in the previous section. We used artificial data, given the absence of real, accurate information of terrorist web-browsing data and counterterrorism strategies. Therefore, we gave value to each of the evaluations that the defender must make about their own decisions and their beliefs about the adversary’s decisions.

We also illustrate the proposed decision model with an example that serves to show some of the computational subtleties, as well as the typical problem solving approach used in a real case. In fact, it may serve as a template for real problems, which would basically add modeling and computational complexities. Essentially, first we structured the problem, then modeled the defender’s evaluations about themselves, and, afterwards, about the adversary. In the computational phase, we simulated the adversary’s problem to obtain their attack probabilities and fed them into the defender’s problem to obtain the optimal defense. Finally, we carried out a sensitivity analysis. For purposes of completeness and comparison, we also provide a standard game-theoretic approach under assumptions of common knowledge.

### 6.1. Structure of the Problem

We begin by identifying the available resources for both the defender and the adversary.

*Defensive resources*. We considered the defender’s defensive resources to be the use of the automatic threat detection system with $d_{1}=1$. Otherwise, $d_{1}=0$.

*Adversarial resources*. For the adversary’s resources, we took the presence of the adversary in the set of monitored sites, with a=1. Otherwise, a=0.

*Results of the game*. Finally, we must consider the results of the decisions of both agents. We assumed that the states of $S_{1}$ and $S_{2}\;$ are 0 or 1, which means, respectively, the success or failure of the detection in terms of the alarm signal of the automatic system (recall that, if $d_{1}=0$, then $s_{1}=0$) and the final detection of the threat after a manual investigation.

### 6.2. The Defender’s Evaluations

Now we consider the evaluations of the beliefs and preferences for the agency, that is, $p_{D}\left(S_{1}|a,d_{1}\right)$, $p_{D}\left(D_{2}|d_{1},s_{1}\right)$, $p_{D}\left(S_{2}|d_{2},a\right)$ and $u_{D}\left(d_{2},s_{2}\right)$, defined in Section 5. In the assumed scenario, none of them require strategic thinking. In the evaluations, we used the different parameters of the online surveillance problem defined in Section 3. Next, we examined the evaluation of the probability distributions involved as well as the utility model.

- *Evaluating*$p_{D}\left(S_{1}|a,d_{1}\right)$. $S_{1}$ represents the probability that the automatic threat detection system generates an alarm, whether there is suspicion or not. Obviously, if the system is not used, it is impossible that it generates an alarm. We established a range of values for this probability, although the defender will operate their problem with the base values. These considerations are reflected in Table 3.
- *Evaluating*$p_{D}\left(D_{2}|d_{1},s_{1}\right)$. $D_{2}$ represents the probability of manually investigating a profile collected both when the automatic system is used and when it is not. We also established a range of values that includes the base value for the defender’s problem, as shown in Table 4.
- *Evaluating*$p_{D}\left(S_{2}|d_{2},a\right)$. $S_{2}$ represents the final success/failure of the surveillance. As described in Section 3, manual investigation was considered 100% effective in confirming or ruling out a threat. In this case, we did not use a range for the values of this probability. Table 5 shows these considerations.
- *Evaluating*$u_{D}\left(d_{2},s_{2}\right)$. Finally, the utility $u_{D}\left(d_{2},s_{2}\right)$ as a measure of the quality of the model. We opted for an exponential utility function that allowed us to order the costs $v_{D}$ of the defender while assuming their (constant) risk aversion. Accordingly, we define $u_{D}\left(d_{2},s_{2}\right)=-\exp\left(c_{D}v_{D}\right)$, with $c_{D}∼U\left(0,\;3\right)$ and consider the parameters shown in Table 6.

### 6.3. The Defender’s Evaluations about the Adversary

The security agency also needs to evaluate $p_{D}\left(A|d_{1}\right)$. This requires strategic thinking, as explained in Section 3. To do this, we must put ourselves in the adversary’s shoes and make assessments about their probabilities and utilities, from the defender’s perspective. Next, we go through how to estimate the probability distributions of the problem at hand and the adversary’s utility function.

- *Evaluating*$P_{A}\left(S_{1}|a,d_{1}\right)$. We assumed that $p_{A}\left(S_{1}=1|d_{1},a\right)$ is similar to $p_{D}\left(S_{1}=1|d_{1},a\right)$. To model our lack of knowledge about the probabilities used by the adversary in their decision problem, we added some uncertainty. In particular, we assumed that, except in cases where $p_{D}\left(S_{1}=1|d_{1},a\right)$ is 0 or 1, for those who suppose that the adversary’s probabilities will match their beliefs $P_{A}$ about $p_{A}\left(S_{1}=1|d_{1},a\right)$ are uniform within the ranges $\left[p_{A}^{\min},p_{A}^{\max}\right]$ of Table 7, evaluated by the defender.
  Then, we modeled $p_{A}$ as a uniform distribution between $p_{A}^{\min}$ and $p_{A}^{\max}$. Thus, $P_{A}\left(S_{1}|a,d_{1}\right)$, was defined by the expression
  $$p_{A}=p_{D}^{\min}+\omega \left(p_{D}^{\max}-\;p_{D}^{\min}\right),$$
  with $\omega ∼U\left(0,1\right)$, so that the uncertainty about ω induced uncertainty about $p_{A}$ to provide $P_{A}$.
- *Evaluating*$P_{A}\left(D_{2}|d_{1},s_{1}\right)$. We adopted the same approach as before, now based on Table 8.
- *Evaluating*PAS2S2d2,ad2,a. We adopted the same approach as before, now based on Table 9.
- *Evaluating*$U_{A}\left(a,s_{2}\right)$. Finally, for utility $u_{A}\left(a,s_{2}\right)$, we also opted for an exponential utility function that allowed us to order the adversary’s costs $v_{A}$, while we assumed their (constant) risk seeking in relation to their benefits. Thus, we defined $u_{A}\left(a,s_{2}\right)=\exp\left(c_{A}\;v_{A}\right)$, with $c_{A}∼U\left(0,\;0.025\right)$ and consider the parameters shown in Table 10.

### 6.4. Results

We solved the problem with the open-source software R (R version 3.3.3 (2017-03-06)) with an Intel^®^ Core${}^{\mathrm{TM}}$ processor i3-2370 CPU at 2.4 GHz, 4Gb RAM on a Windows 10 64-bit operating system. In our example, the computation time was acceptable (15–20 s per problem on average) and therefore we did not consider the implementation and its performance as the object of the analysis. In any case, it should be noted that the resolution of the problem implied a Monte Carlo simulation for each value $d_{1}$ and that in each simulation we must propagate uncertainty at different levels, which became a strong computational challenge for larger problems.

For comparative and sensitivity analysis purposes, we prepared an experiment with 1000 random scenarios for five levels $c_{D}$ of risk aversion of the defender, specifically $c_{D}=\left(0.01,\;0.1,\;0.5,\;1.0,\;3.0\right)$. In total, we obtained a set of 5000 solutions. In this way, we intended to determine how the parameters of the problem influenced the defender’s optimal decision $d_{1}^{*}$ and at the same time compared their behavior according to their level of risk aversion, between $c_{D}=0.01$ (minimum) and $c_{D}=3.0$ (maximum). In Table 11, we include the parameters that we used when the 1000 scenarios were generated for each value of $c_{D}$.

The implementation of our evaluation model allowed us to obtain in each run (i) the optimal solution $d_{1}^{*}$ for the defender; and (ii) the conditional probability $p_{D}\left(A|d_{1}\right)$ that the defender needed to solve their problem. As explained above, this probability was estimated using a Monte Carlo simulation with n=10,000 replications for each value $d_{1}\in D$. Table 12 and Table 13 show an extract of the results for one scenario and different levels of $c_{D}$ of risk aversion of the defender, and the explicit form of the probability $p_{D}\left(A|d_{1}\right)$ for that scenario.

Thus, for example, the probability that the adversary is present in the set of monitored websites, taking into account the possibility that the defender is monitoring their navigation, is p^DA=1A=1d1=1d1=1=0.91. This means that, in this example, solving the defender’s problem ends with the optimal solution $d_{1}^{*}=1$ for levels $c_{D}$ of the defender’s risk aversion 0.01 and 0.10 and the contrary for higher levels.

**Figure 8:**
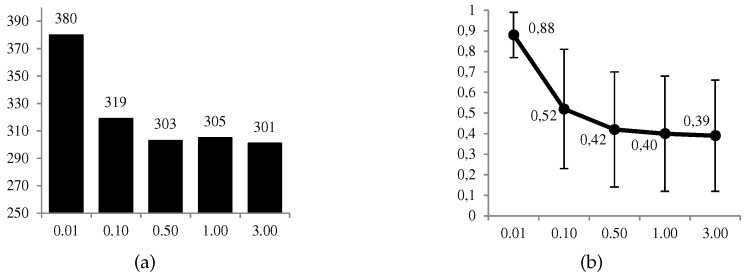
Results of ARA. (**a**) No. of cases (over 1 000) and (**b**) ratio $\frac{\psi_{D}\left(d_{1}^{*ARA}\right)}{\psi_{D}\left(d_{1}^{\neg *}\right)}$ (average ± deviation) for $d_{1}^{*}$, both depending on the risk aversion level $c_{D}$ of the defender (abscissa axis).

**Figure 9:**
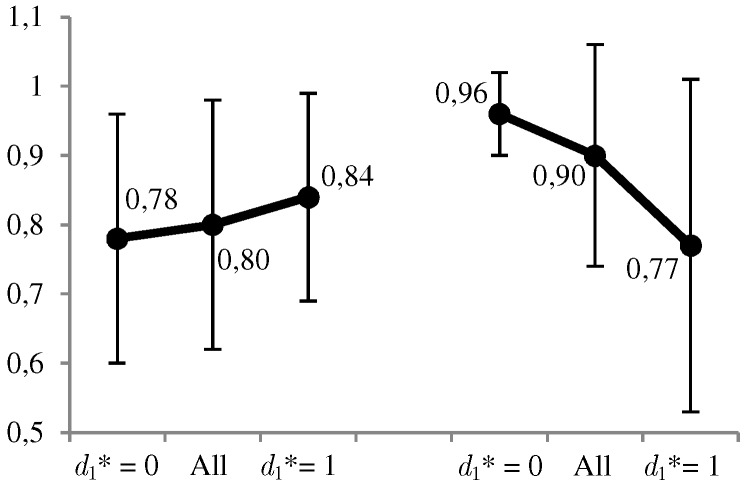
Values of ${\hat{p}}_{D}\left(A|d_{1}=1\right)$. On the left if $d_{1}=1$ and on the right if $d_{1}=0$.

In Figure 8 and Figure 9, we can observe graphically some of the most relevant results. The favorable use of the system, $d_{1}^{*}=1$, is given in a moderate proportion of the 1000 cases (between 30% and 38%), and in a more conservative way at a higher level $c_{D}$ of the defender’s risk aversion. The same decreasing behavior is observed for the ratio between the expected utilities of the optimal solution and its opposite. On the other hand, we have the average values of the estimates, p^DA=1A=1d1d1, for which we observe that p^DA=1A=1d1=1d1=1⩾p^DA=1A=1d1=0d1=0 when $d_{1}^{*}=1$, and conversely in the opposite case. All these results confirm what we intuitively assumed a priori, and the correct behavior of the calculations.

Finally, we adjusted a parametric model to the set of solutions, specifically a logistic regression of the form $logit\left(d_{1}^{*}\right)=b_{0}+b_{1}x_{1}+b_{1}x_{1}+\cdots +b_{i}x_{i}$, with the aim of determining the relationship between $d_{1}^{*}$ and the parameters of the problem. To select the best model, we used the bestglm (Available online: https://cran.r-project.org/web/packages/bestglm/index.html (accessed on 8 May 2012)) package of R. The reason was to avoid losing information and overestimating the logit model. Table 14 shows the results of the adjustments, where between the null model and the complete model, the best model obtained is “ARA08.06” (logit model with six variables out of eight available variables, highlighted in bold). Thus, the model indicates that, a priori, we could do without the parameters $\beta^{\mathrm{base}}$ and λ to explain the optimal decision ${\;d}_{1}^{*}=1$ of the defender, while the parameter ${\;\rho }^{\mathrm{base}}$ (proportion of profiles investigated manually when the system is not used), with an oddsratio=16.56, is shown to be highly influential.

The confusion matrix (The cut-off value is 0.40 for a dataset with 1608 reference cases over 5000) shown in Table 15, computed from the predictions of the “ARA08.06” model, indicates that 3703 of the 5000 scenarios (74%) would be correctly predicted, with 953 out of 1 608 (59%) referring to the cases where $d_{1}^{*}=1$.

At this point, we want to highlight that other parametric and/or nonparametric adjustments can be used alternatively to logistic regression. It is also possible to analyze the sensitivity of the problem from other angles, be it for example through game theory in its classical form or through differential calculus, to find solutions and optimal parameter configurations. We look at this in the following subsection, which analyzes the problem from a standard game-theoretic approach.

#### Comparison with Game Theory

We next solved our example using the game theory approach and compared the results with the solution offered by ARA. The standard game theory approaches generally assume a common knowledge about the structure of the game (values at stake, resources available for the players, feasible assignments, etc.) as well as the utilities and probabilities of the players. In addition, the existence of objective probabilities for uncertainties is also usually assumed, in our case the results of automatic and/or manual investigations, $S_{1}|d_{1},a$ and $S_{2}|a,d_{2}$.

We assume that the conditional probabilities $\left(S_{1}|d_{1},a\right)$ and $p(S_{2}|a,d_{2})$ derive, respectively, from $p_{D}\left(S_{1}|d_{1},a\right)$ and $p_{D}\left(S_{2}|a,d_{2}\right)$, that is, the defender’s belief about the probability that the adversary’s presence is not detected. These probabilities now represent objective non-detection probabilities, and both the defender and the adversary know them. In addition, the assumption of common knowledge ensures that the defender knows the probabilities used by the adversary when they solve their decision problem, and therefore does not need to represent the uncertainty surrounding them.

To resolve the problem we adapted the reduction algorithm of influence diagrams proposed by [27] to evaluate an influence diagram that represents a decision problem of a single agent, to solve the sequential defense–attack games formulated as multi-agent influence diagrams. We solved, in parallel, the experiment proposed for ARA when the coefficients of risk aversion and risk seeking for the defender and the adversary are, respectively, $c_{D}=\left(0.01,\;0.1,\;0.5,\;1.0,\;3.0\right)$ and $c_{A}=0.0125$ (remember that originally $c_{A}∼U\left(0,\;0.025\right))$. These values determine the utility functions of the defender and the adversary, given, respectively by $u_{D}\left(d_{2},s_{2}\right)$ and $u_{A}\left(a,s_{2}\right)$, which are also common knowledge. In general, we expect coincidental and opposite results that reveal the different assumptions of the methods. In Table 16, we show the same extract of results that Table 12 shows obtained by ARA, now adding the optimal solution provided by game theory. In this scenario and depending on the risk aversion level $c_{D}$ of the defender, ARA behaves more prudently than game theory, which constantly obtains the same solution $d_{1}^{*GT}=1$.

**Figure 10:**
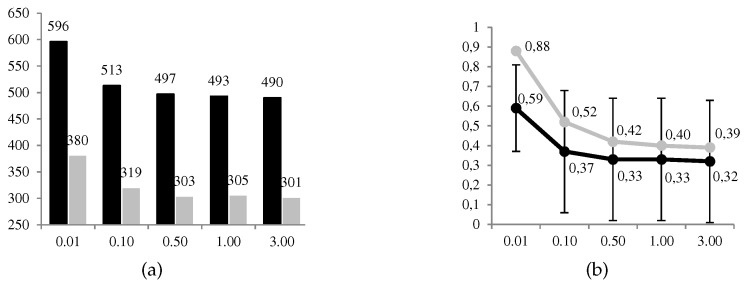
ARA results (grey) vs. game theory (black). (**a**) No. of cases (over 1000) and (**b**) ratio $\frac{\psi_{D}\left(d_{1}^{*GT}\right)}{\psi_{D}\left(d_{1}^{\neg *}\right)}$ (average ± deviation) for $d_{1}^{*}=1$, both depending on the level $c_{D}$ of risk aversion of the defender.

In Figure 10, we can observe graphically some of the results obtained. The favorable use of the system, $d_{1}^{*}=1$, is given in a proportion between 49% and 59% of 1000 cases, decreasing to a greater level $c_{D}$ of risk aversion of the defender. The same decreasing behavior is observed for the ratio between the expected utilities of the optimal solution and its opposite. Compared to ARA, the frequency of favorable use of the system is significantly less conservative. This result satisfies us because it corresponds to ARA applications solving other problems. The opposite occurs with the ratio of the expected utilities between the optimal solution and the opposite, where ARA also has decreasing but higher ratios. We understand that these differences are found in the terms used to calculate the expected utilities of the defender in node A.

In Table 17, we show the same logistic adjustment used for the results obtained with ARA. Between the null model and the complete model, the best model obtained with game theory is “GT08.05” (logit model with five variables out of eight available variables). The model indicates that, a priori, we could dispense with parameters $\alpha^{\mathrm{base}}$, ϕ and λ to explain the defender’s optimal decision, while parameter $\beta^{\mathrm{base}}$ (false positives), with an oddsratioof29.88, demonstrates to be highly influential. For comparison, we also include in the table the best ARA model obtained, “ARA08.06”.

The confusion matrix (The cut-off value is 0.55 for a dataset with 2589 reference cases over 5000) shown in Table 18, computed from the predictions of the “GT08.05” model, indicates that 3067 of the 5000 scenarios (61%, 74% with ARA) would be correctly predicted, 1312 out of 2589 (51%, 59% with ARA) referring to the cases in which $d_{1}^{*}=1$.

In general, we can conclude that the results obtained with ARA are more satisfactory than those obtained with game theory. In contrast to standard game theory, however, ARA provides more costly solutions from a computational point of view, but more realistic in terms of the dynamics and perspective of the game proposed between adversaries to solve the problem of online surveillance.

## 7. Discussion

We defined the problem of online surveillance based on the intrusion-detection problem posed by [18] through backward induction. The choice of methodology and model applied to this problem is one of the central ideas of this work. In this sense, we believe that the essence of the problem is adequate for the use of ARA, which arises, among other aspects, from (i) the need to address an efficient allocation of security resources for managing a terrorist threat and (ii) to improve the methods of decision analysis when the risks are derived from intentional actions by intelligent adversaries. These improvements of ARA are aimed to address the unrealistic situations such as the hypothesis of common knowledge about payments and probabilities among adversaries that the classical game theory requires, and the unpredictability of the adversaries’ intentions that the standard risk analysis supposes.

On this basis, we investigated an ARA model as a novel way of proposing and solving the problem of online surveillance, where, among other aspects, we do not assume the hypothesis of common knowledge. Unlike the problem tackled by [18], we evaluated the problem of online surveillance faced by a security agency that monitors a set of specific websites by tracking and classifying profiles that are potentially suspected of carrying out terrorist attacks.

Our analysis constitutes a preliminary, theoretical step in that it aimed to establish a point of departure and connection between the analytical framework provided by ARA, a young field within risk analysis, and the problem of online surveillance with counterterrorist purposes, understood as a game between opponents who wanted to maximize their benefits.

To give consistency to our proposal, we have illustrated a feasible architecture for online surveillance based on an engine for tracking user navigation traces on monitored websites and an automatic classifier of suspects, thought of as a classification method based on artificial intelligence. However, we recognize that the implementation of such a surveillance infrastructure may pose several technical difficulties. A number of security and privacy aspects, out of the scope of this work, would need to be considered if such an infrastructure were to be implemented in real practice; among those aspects, special attention should be paid to the exchange of information between the security agency and the tracking/advertising platform(s) and the design of secure authentication schemes [28,29,30].

In this scenario, we evaluated the adoption of automatic technology compared to the status quo that involves the manual investigation of profiles. The automatic threat detection system, nonetheless, is limited to the extent given by the sensitivity and specificity parameters of the artificial-intelligence algorithm responsible for classifying the collected profiles. Specifically, the compromise between the defined parameters and the evaluations of the probabilities and payments of the agents governed by the dynamic strategy modeled with ARA determines the limitations of the automatic system. The results of our experiments indicate that the use of the automatic detection system is strongly influenced by the proportion of profiles that the security agency could investigate manually (status quo case). This is a proportion that supposedly depends in turn on the budget available for intelligence analysts.

We applied the ARA methodology, which offers the possibility of treating the problem with game theory and risk analysis approaches in a new perspective of decision analysis against intelligent adversaries, who increase the risk of security and uncertain results.

Our experimental results corroborate the benefits of the proposed model and at the same time are indicative of its potentialities.

Compared to the analysis of the problem from standard game theory, ARA indicated in general greater prudence in the deployment of the automatic system, even more at higher levels of risk aversion. In addition, the behavior of estimated conditional probabilities correctly responded to our intuitions (${\hat{p}}_{D}\left(A=1|d_{1}=1\right)⩾{\hat{p}}_{D}\left(A=1|d_{1}=0\right)$ when $d_{1}^{*}=1$, and conversely in the opposite case). From this point of view, the ARA model is more attractive than the standard game-theoretic model since it behaves satisfactorily without having to relax crucial hypotheses such as common knowledge and therefore subtracting realism from the problem.

We used a parametric model with the aim of understanding the relationship between the optimal decision $d_{1}^{*}=1$ and the parameters of the problem. This can be a way of not having to execute the resolution algorithm countless times to determine the adjustment of the system parameters. This and other decisions can be part of the implementation of the online monitoring architecture (machine learning, sensitivity analysis, etc.). In addition to the better parametric adjustment of ARA over standard game theory, we observed that $\rho^{\mathrm{base}}$ (proportion of profiles investigated manually in case of not using the system) is determinant for ARA while $\beta^{\mathrm{base}}$ (false positives) was for game theory. $\beta^{\mathrm{base}}$ depends on how good our detection system was and $\rho^{\mathrm{base}}$ on the available resources. A priori the two implications could be coherent and a further debate could explore this, for example, depending on how the surveillance architecture is implemented.

Given the absence of real, accurate information of terrorist web-browsing data, our experiments were conducted on artificial data. We consider that the obtained results, although based on artificial and therefore limited data, place us positively at this starting point, as they have been satisfactorily contrasted with standard game theory. The obtained experimental results are solvent from a theoretical standpoint. Furthermore, from a practical point of view, those results provide confirmatory evidence as we could corroborate some previous intuitions we expected from ARA, as illustrated in Figure 8 and Figure 9, particularly with regard to the predictions about the attacker $p_{D}\left(A|d_{1}\right)$, whose evaluation is key for ARA, and which we derived from our uncertainty about the adversary’s problem and under the hypothesis that the adversaries endeavor to maximize their expected utility. The following support the validity and appropriateness of the proposed approach: (i) the model formulation used in this work is one of the basic template models employed in counterterrorism; (ii) this model has been successfully addressed by ARA; and (iii) ARA is a robust, extensively investigated theoretical framework in the literature [8,21,31].

Furthermore, ARA goes beyond the dynamics of a player facing “nature”, introducing in its place intelligent adversaries in a game of rational confrontation represented by the agents’ utility functions. In addition, another important advantage of ARA is its use of multi-agent influence diagrams, a graphic tool that allows clearly representing a decision problem between more than one agent. In a nutshell, we can conclude that ARA is an excellent option for modeling and solving the problem posed against the classical model of game theory.

## 8. Conclusions and Future Work

In recent years, Western countries have allocated tremendous amounts of resources to fight terrorism. As in any war, the battle occurs in various environments and the Internet, with the advent of the IoT, is one of the most powerful ones for propagating a threat and recruiting terrorists. However, at the very same time, this environment is the perfect storm for the development of ubiquitous online surveillance.

In this work, we first examined the suitability of standard game theory and ARA, to tackle the online surveillance problem in which a security agency aims at countering terrorism online by deploying an automatic threat detection system on certain target websites. Then, we proposed an ARA-based model to analyze the feasibility of using such an automatic system, and determined under which conditions said deployment is better than the traditional model in which terrorist online activity is inspected manually by agents. Experimental results show that our ARA-based model is more attractive than the standard game-theoretic model as the former behaves satisfactorily without having to relax crucial hypotheses such as common knowledge and thus subtracting realism from the problem. Specifically, experiments on artificial data showed ARA would correctly predict 74% of the 5000 simulated scenarios, 59% of them corresponding to the case $d_{1}^{*}=1$. In contrast, GT would yield 61% correct prediction, with 51% of the analyzed scenarios corresponding to $d_{1}^{*}=1$.

Future research lines include adopting other sequences and/or introducing new intermediate decisions to be taken into account (for example, changing the uncertainty node D2 to a decision node). For this, we can use other ARA templates and model new situations, in both sequential and simultaneous game dynamics.

## Figures and Tables

**Table 1:** Parameters of the online surveillance problem.

Symbol	Description
α	Probability of ASC alarm due to suspicion (true positive)
β	Probability of ASC alarm without suspicion (false positive)
π	Probability of presence of a suspicious user
ρ	Probability of manual investigation without using ASC
$\rho_{1}$	Probability of manual investigation when the ASC generates an alarm
$\rho_{0}$	Probability of manual investigation when the ASC does not generate an alarm
*c*	Cost of manual investigation; c⩽ϕd, ϕ⩽1
*d*	Damage derived from an undetected suspect
ϕ	Cost/damage coefficient of the system
*b*	Benefit for suspects not detected; l⩾(1+λ)b,λ⩽1
*l*	Loss for suspects not detected
λ	Benefit/loss coefficient of the suspect

**Table 2:** Comparison between standard game theory and ARA.

Requirements	Standard Game Theory	ARA
opponents aim to maximize their utility	**✓**	**✓**
uncertainty about the attacker’s actions	**✗**	**✓**
incomplete information about the evaluation of the objectives between opponents	**✗**	**✓**
simultaneous and sequential decisions	**✓**	**✓**

**Table 3:** $p_{D}\left(S_{1}=1|d_{1},a\right)$.

	a=1	a=0
$d_{1}=1$	$\alpha^{\mathrm{base}}$	$\beta^{\mathrm{base}}$
$d_{1}=0$	0	0

**Table 4:** $p_{D}\left(D_{2}=1|d_{1},s_{1}\right)$.

	$s_{1}=1$	$s_{1}=0$
$d_{1}=1$	$\rho_{1}^{\mathrm{base}}$	$\rho_{0}^{\mathrm{base}}$
$d_{1}=0$	$\rho^{\mathrm{base}}$	

**Table 5:** $p_{D}\left(S_{2}=1|a,d_{2}\right)$.

	a=1	a=0
$d_{2}=1$	1	0
$d_{2}=0$	0	0

**Table 6:** $v_{D}\left(d_{2},s_{2}\right)$.

	$s_{2}=1$	$s_{2}=0$
$d_{2}=1$	*c*	c+d
$d_{2}=0$	0	*d*

**Table 7:** $p_{A}\left(S_{1}=1|d_{1},a\right)$.

	a=1	a=0
$d_{1}=1$	$\left[\alpha^{\min},\alpha^{\max}\right]$	$\left[\beta^{\min},\beta^{\max}\right]$
$d_{1}=0$	0	0

**Table 8:** $p_{D}\left(D_{2}=1|d_{1},s_{1}\right)$.

	$s_{1}=1$	$s_{1}=0$
$d_{1}=1$	$\left[\rho_{1}^{\min},\rho_{1}^{\max}\right]$	$\left[\rho_{0}^{\min},\rho_{0}^{\max}\right]$
$d_{1}=0$	$\left[\rho^{\min},\rho^{\max}\right]$	

**Table 9:** $p_{D}\left(S_{2}=1|a,d_{2}\right)$.

	a=1	a=0
$d_{2}=1$	1	0
$d_{2}=0$	0	0

**Table 10:** $v_{A}\left(a,s_{2}\right)$.

	a=1	a=0
$d_{2}=1$	b−l	*b*
$d_{2}=0$	0	0

**Table 11:** Main parameters of the experimental evaluation.

Sensitivity and Specificity		Proportion of Manual Investigations	Costs and Coefficients	
$\alpha^{\mathrm{base}}$	$\beta^{\mathrm{base}}$	$\rho^{\mathrm{base}}$, $\rho_{1}^{\mathrm{base}}$,$\;\rho_{0}^{\mathrm{base}}$	ϕ	λ
U(0.60,0.99)	$U\left(0,\;0.1\right)$	U(0,1)	U(0,1)	U(0,1)
$\alpha^{\min}$	$\beta^{\min}$	$\rho^{\min}$, $\rho_{1}^{\min}$,$\;\rho_{0}^{\min}$	*c*	*b*
$U(0.60,\;\alpha^{\mathrm{base}})$	$U(0,\;\beta^{\mathrm{base}})$	$U(0,\;\rho^{\mathrm{base}})$ ditto for $\rho_{1}^{\min}$ and $\rho_{0}^{\min}$	ϕd ϕ∼U(0,1)	100
$\alpha^{\max}$	$\beta^{\max}$	$\rho^{\max}$, $\rho_{1}^{\max}$,$\;\rho_{0}^{\max}$	*d*	*l*
$U(\alpha^{\mathrm{base}},\;0.99)$	$U(\beta^{\mathrm{base}},0.1)$	$U(\;\rho^{\mathrm{base}},1)$ ditto for $\rho_{1}^{\max}$ and $\rho_{0}^{\max}$	100	l=(1+λ)b λ∼U(0,1)

**Table 12:** Summary of the results obtained for our ARA model.

$d_{1}^{*}$	$c_{D}$	$\frac{\psi_{D}\left(d_{1}^{*}\right)}{\psi_{D}\left(d_{1}^{\neg *}\right)}$	$\alpha^{\mathrm{base}}$	$\beta^{\mathrm{base}}$	$\rho^{\mathrm{base}}$	$\rho_{1}^{\mathrm{base}}$	$\rho_{0}^{\mathrm{base}}$	ϕ	λ	${\hat{p}}_{D}\left(A=1|d_{1}\;\right)$	
										$d_{1}=1$	$d_{1}=0$
1	0.01	0.73	0.92	0.01	0.48	0.89	0.73	0.08	0.05	0.91	0.99
1	0.10	0.53	0.92	0.01	0.48	0.89	0.73	0.08	0.05	0.91	0.99
0	0.50	0.21	0.92	0.01	0.48	0.89	0.73	0.08	0.05	0.91	0.99
0	1.00	0.08	0.92	0.01	0.48	0.89	0.73	0.08	0.05	0.91	0.99
0	3.00	0.08	0.92	0.01	0.48	0.89	0.73	0.08	0.05	0.91	0.99

**Table 13:** Form of ${\hat{p}}_{D}\left(A|d_{1}\right)$.

	a=1	a=0
$d_{1}=1$	0.91	0.09
$d_{1}=0$	0.99	0.01

**Table 14:** Logit models for the ARA results.

Model	cte. Logit	$\alpha^{\mathrm{base}}$	$\beta^{\mathrm{base}}$	$\rho^{\mathrm{base}}$	$\rho_{1}^{\mathrm{base}}$	$\rho_{0}^{\mathrm{base}}$	ϕ	λ	$c_{D}$	AIC
ARA00.00	−0.75	0.00	0.00	0.00	0.00	0.00	0.00	0.00	0.00	6283.70
**ARA08.06**	**0.64**	**−1.49**	**0.00**	**2.81**	**−0.46**	**−2.37**	**−0.58**	**0.00**	**−0.08**	**5281.03**
ARA08.08	0.59	−1.46	−1.08	2.81	−0.47	−2.37	−0.58	0.16	−0.08	5277.06

**Table 15:** Confusion matrix of the “ARA08.06” logit model.

		Pred.	
		$d_{1}^{*}=0$	$d_{1}^{*}=1$
Obs.	$d_{1}^{*}=0$	2753	639
	$d_{1}^{*}=1$	655	953

**Table 16:** Extract of standard game theory (GT) results vs. ARA results.

$c_{D}$	$d_{1}^{*\mathrm{GT}}$	$\frac{\psi_{D}\left(d_{1}^{*\mathrm{GT}}\right)}{\psi_{D}\left(d_{1}^{\neg *}\right)}$	$d_{1}^{*\mathrm{ARA}}$	$\frac{\psi_{D}\left(d_{1}^{*\mathrm{ARA}}\right)}{\psi_{D}\left(d_{1}^{\neg *}\right)}$	$\alpha^{\mathrm{base}}$	$\beta^{\mathrm{base}}$	$\rho^{\mathrm{base}}$	$\rho_{1}^{\mathrm{base}}$	$\rho_{0}^{\mathrm{base}}$	ϕ	λ
0.01	1	0.77	1	0.73	0.92	0.01	0.48	0.89	0.73	0.08	0.05
0.10	1	0.04	1	0.53	0.92	0.01	0.48	0.89	0.73	0.08	0.05
0.50	1	0.04	0	0.21	0.92	0.01	0.48	0.89	0.73	0.08	0.05
1.00	1	0.04	0	0.08	0.92	0.01	0.48	0.89	0.73	0.08	0.05
3.00	1	0.04	0	0.08	0.92	0.01	0.48	0.89	0.73	0.08	0.05

**Table 17:** Logit models for the game theory results.

Model	Cte. Logit	$\alpha^{\mathrm{base}}$	$\beta^{\mathrm{base}}$	$\rho^{\mathrm{base}}$	$\rho_{1}^{\mathrm{base}}$	$\rho_{0}^{\mathrm{base}}$	ϕ	λ	$k_{D}$	AIC
GT00.00	0.07	0.00	0.00	0.00	0.00	0.00	0.00	0.00	0.00	6927.13
**GT 08.05**	**0.34**	**0.00**	**3.40**	**1.34**	**−1.25**	**−0.74**	**0.00**	**0.00**	**−0.09**	**6549.20**
GT08.08	−0.13	0.50	3.28	1.35	−1.24	−0.74	0.05	0.10	−0.09	6550.38
ARA08.06	0.64	−1.49	0.00	2.81	−0.46	−2.37	−0.58	0.00	−0.08	5281.03

**Table 18:** Confusion matrix of the “GT08.05” logit model.

		Pred.	
		$d_{1}^{*}=0$	$d_{1}^{*}=1$
Obs.	$d_{1}^{*}=0$	1595	816
	$d_{1}^{*}=1$	1277	1312
